# Optical Anisotropy of Polyethylene Terephthalate Films Characterized by Spectral Means

**DOI:** 10.3390/polym16060850

**Published:** 2024-03-20

**Authors:** Mihaela Iuliana Avadanei, Dan Gheorghe Dimitriu, Dana Ortansa Dorohoi

**Affiliations:** 1 Petru Poni Institute of Macromolecular Chemistry, 41A Grigore Ghica Voda Alley, RO-700487 Iasi, Romania; mavadanei@icmpp.ro; 2 Faculty of Physics, Alexandru Ioan Cuza University, 11 Carol I Blvd., RO-700506 Iasi, Romania; ddorohoi@uaic.ro

**Keywords:** polyethylene terephthalate, birefringence and its dispersion, ellipsometry, channeled spectra, dichroic ratio, ATR absorbance ratio

## Abstract

Polyethylene terephthalate (PET) films are the subject of intensive research because of great interest in using them in applications, especially in medicine. From an optical point of view, PET films with a low degree of stretching can be considered uniaxial materials, for which the determination of the linear birefringence and its dispersion is very important. Two methods were applied here for the estimation of these parameters: the ellipsometric method and the channeled spectra method. The ellipsometric method uses monochromatic radiation; therefore, the linear birefringence of the PET films is determined for a given value of the radiation wavelength. The channeled spectra method allows for the estimation of the linear birefringence and its dispersion for a large range of wavelengths in the visible spectrum. A decrease in both parameters with the increase in the wavelength was recorded. To evidence the microstructure of PET films and the conformational changes induced by elongation and to evaluate the degree of orientation, a polarized infrared spectral study in attenuated total reflection (ATR-FTIR) mode was performed. The dichroic ratio (between the absorbance measured with linearly polarized radiation parallel and orthogonal relative to the stretching direction, respectively) and the ATR absorbance ratio for the machine direction (MD) and transversal direction (TD) configurations both for the stretched and unstretched PET samples were measured.

## 1. Introduction

Polyethylene terephthalate (PET) is a thermoplastic polymer, a polyester with a partially aliphatic and aromatic structure, that can be amorphous or semi-crystalline [1], made starting from ethylene and paraxylene, whose derivatives, i.e., ethylene glycol (a diol) and terephthalic acid (a dicarboxylic acid) react in the presence of a catalyst to obtain the PET resin in the shape of long polymer chains. They can be subsequently further stretched by means of several techniques, which improve their crystallinity by accentuating the reciprocal orientation of the molecules, and thus change their properties, not only the optical ones but also others, e.g., the thermal stability improves, but the transparency decreases [2,3].

Commercial PET has a density of 1.41 g/cm^3^, a breaking strength of 50 MPa, a Young’s modulus of 1700 MPa, a melting temperature of 265 °C, and a glass transition temperature between 69 and 115 °C, above which PET is highly stiff [4].

Other key features of PET are its low production cost and recyclability [3,4]. Various preparation methods are available for PET films: casting, blowing, and extrusion. The as-prepared PET can be post-processed via surface modifications, printing, and lamination in order to be used for various applications. The preparation and post-processing parameters can be used to control the properties of the resulting PET. As recyclability is concerned, PET is the most recyclable plastic to date, with three groups of methods: primary methods or mechanical ones, secondary methods, namely chemical ones, and tertiary recycling, consisting of the recovery of energy content from the plastic waste by incineration [4].

Originally produced for synthetic fibers in the 1930s, PET also started to be used for film packaging, and then, in the early 1970s, the biaxially oriented bottle-blowing technique was commercially developed. The strength of the material caused PET to become successful. Thus, in the industry of carbonated soft drinks, the pressure inside the bottle can reach up to 600 kPa, but PET can stand such a high pressure due to the alignment of macromolecules after stretching the material during the technological process of film manufacturing and bottle shaping [5]. The same property, along with stiffness, determined the use of PET for fiber and textile production, including carpets. Except for its strength, there are other properties of PET films that make them useful for various applications, especially for packaging and electrical insulation. PET has high chemical resistance to acids, bases, and solvents, making it suitable in harsh chemical environments. Also, moisture, gases, and other environmental factors are stopped by PET films acting as barriers. Along with the latter property, its high transparency (above 90%) made it popular for food packaging and consumer electronics. The same transparency led to the use of PET films as a laminated protective layer against environmental hazards for flexible electroluminescence devices [6].

PET is thermally stable up to high temperatures; therefore, it can be easily thermally processed. Two features make it very useful. One refers to its biocompatibility, and thus, it can be used for various medical applications since it can come in contact with living tissues without inducing any negative reaction. PET films can be used not only for medical packaging and sterilization wraps (given their transparency, barrier properties, chemical attack, and high temperature resistance, including those during the sterilization processes) but also for medical implants as coatings, such as for cardiovascular prostheses [7,8,9,10] and blood vessels’ metallic stents [11], especially since it was proved that PET has the ability to reduce thrombus formation [12,13]. Still, microorganisms (e.g., bacteria and viruses) can proliferate on the surface of PET films, but the problem was solved in more than one way. A chitosan layer was coated on the PET surface, a coating that was then loaded with silver nanoparticles that are well known for their antiseptic properties [14]. Also, chitosan is a polymer that exhibits efficient antimicrobial activity against different pathogenic microorganisms [14,15]. Another method for avoiding the proliferation of microorganisms on PET is the functionalization of its surface by means of gas and/or plasma exposure, leading to a more hydrophilic or hydrophobic surface [15,16]. Thus, it was found that fluorinated PET films have a higher surface hydrophilicity than unfluorinated PET films and also that the hydrophilicity increases with fluorination time due to the number increase in polar groups in the fluorinated molecules that repel water [16]. Fluorination is also used to improve PET surface concerning its low surface free energy and high chemical inertness, disadvantages inducing poor wettability, printability, and adhesion of PET films, requesting solutions to overcome these issues [17].

In dentistry, studies have shown that PET surface modification improves cell biocompatibility, increases cell production and adhesion, and enhances osseointegration [18]. The intensification of osseointegration has been verified in the case of PET when associated with other compounds, such as hydroxypropylcellulose (HPC) or hyaluronic acid, which, when added to the surface of the polymer, firstly favors the formation of collagen [19,20]. Moreover, pure PET manifests resistance to dye staining, thus being recommended as dental material [18].

Among the multiple applications, polyethylene terephthalate is used as a nanoporous membrane material for polymeric nuclear track detectors [21,22] or for the detection of heavy metal ions in water [23]. Also, transparent PET substrates with low haze and high optical smoothness are ideal for applications in touch panels and solar cells [24,25]. Conductors such as indium tin oxide (ITO) [26] and silver nanoparticles [27] were deposited onto PET substrates, obtaining films with suitable properties for applications in flexible displays, integrated circuits, or optoelectronic devices.

For PET films, the draw ratio, namely the ratio of the final film thickness to the initial film thickness after the film has been stretched/drawn, determines most of the properties of the resulting film, including its mechanical strength, optical transparency, and thermal stability. In general, the draw ratio of PET films can be controlled by adjusting the stretching temperature, stretching rate, film orientation (i.e., uniaxial stretching typically yields higher draw ratios compared to biaxial stretching), and chemical composition (e.g., additives and copolymers) in order to produce films with specific properties, such as high mechanical strength or improved optical clarity [28].

Birefringence is the property of a material that exhibits two different indices of refraction for light polarized in different directions, a property which can be observed as a double refraction of light when passing through the material, leading to the splitting of a single incident light beam into two separate beams that are polarized in perpendicular directions. The birefringence degree of a material, computed as the difference between the two refractive indexes of polarized refracted/transmitted radiation, depends on the molecular structure of the substance and on the wavelength of light. Highly ordered materials, such as crystals or stretched molecular structures, have higher levels of birefringence than materials with disordered structures, such as amorphous materials, and thus, birefringence measurements can be used for the structural characterization of materials. PET layers are characterized by a greater value of birefringence in the range of shorter wavelengths compared with that one corresponding to higher values of wavelength. The wavelength dependence of birefringence for PET films is generally minimal. Birefringence is sometimes undesirable, while other times is useful. Thus, the birefringence of PET films makes them useful for polarizing filters, LCD displays, and optical fibers.

For PET films, it was found that if uniaxial stretched, the refractive index corresponding to the tensile direction changes significantly as compared to the refractive index corresponding to the direction perpendicular to the tensile stress, while the birefringence exhibits a linear increase with strain [29,30]. This happens because large orientation effects were obtained in the stretching direction [31] due to PET’s ability to crystallize at large draw ratios during the deformation process [32]. In this way, an anisotropic structure of PET can be obtained, usually used in polarization control devices [33].

The polymeric chains mostly lie in-plane during the fabricating process [34] and a supplementary applied uniaxial tension has a slight impact on the structure of the molecular chains situated in the out-of-plane direction [29]. In this way, the out-of-plane optical parameters are supposed to remain unchanged and only the in-plane optical properties modifications along the stretching direction are considered [29,30]. According to this, and due to the very close values of the two extraordinary refractive indices [35], it can be considered that, from an optical point of view, PET films act as uniaxial materials, and there is a single direction governing the optical anisotropy. This direction is called the optical axis of the material, and it coincides with the main axis of stress [34].

Birefringence can be measured using various techniques: optical microscopy using cross-polarized light, interference techniques—measurement of the phase difference between two polarized components of light that pass through a birefringent material, ellipsometry [29,30,34,35,36,37,38,39]. The latter allows for measuring the change in polarization of light as it passes through a material. By measuring the changes in polarization, the birefringence of the material can be determined, along with its thickness. Ellipsometric measurements consist of sending a polarized beam onto the sample and analyzing the polarization degree of the reflected light. Such a technique is nondestructive, and it is widely used in optical engineering, materials science, and semiconductor industry.

The aim of this research was to estimate the birefringence and its dispersion in visible range by two methods, namely the ellipsometric method and the channeled spectra method, for PET films with different degrees of stretching corroborated the microstructure of PET films and the orientation of conformers in PET samples. The polarized ATR-FTIR analysis allowed us to determine the dichroism, D (the ratio between the absorbance measured with linearly polarized radiation parallel and orthogonal relative to the stretching direction), and the ATR absorbance ratio for the machine direction (MD) and transversal direction (TD) configurations both for the stretched and unstretched PET samples.

This study characterizes the anisotropic properties of the commercial PET samples and demonstrates the applicability of the new method based on ellipsometric measurements of the birefringence, recently applied to polyvinyl alcohol (PVA) films [39].

## 2. Materials and Methods

The studied PET is from a roll of commercial origin (Terom, Iasi, Romania, with the following specifications: *T_g_* = 80 ± 2 °C, 40% crystallinity, 1.38 g/cm^3^ density) of 75 μm thickness. The surface contaminants were removed by successive washings with acetone and double distilled water.

Small samples of 1.0 × 1.5 and 5 × 3 cm^2^ size were cut and uniaxially stretched (the long side of the sample parallel to the stretching direction) with the help of a lab-made machine (that also assures the heating of the sample during stretching).

When the polymer layers are stretched, the alignment of the polymer chains is enhanced, and the difference between their optical parameters increases. The linear birefringence of the stretched polymer layers is composed of an intrinsic one and one induced by stretching, called orientation birefringence. The stretching direction coincides with the optical axis of the polymer layer.

**Figure 1:**
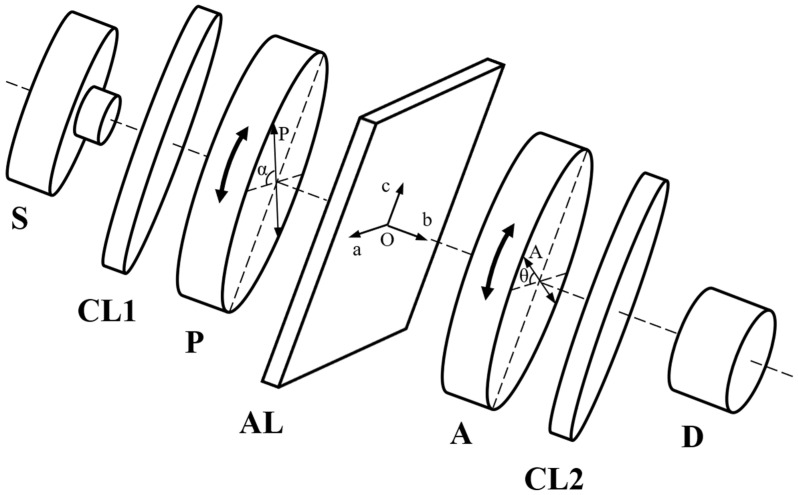
Schematic representation of the device used to establish the birefringence of the anisotropic later (stretched PET films). S—light source, CL1, and CL2—converging lens, P and A—polarizing filters with the role of polarizer and analyzer, respectively, AL—anisotropic layer, D—detector, *α*—azimuth angle between the transmission direction of the polarizing filter P and the principal axis O*a* of the anisotropic layer, *θ*—azimuth angle between the transmission direction of the polarizing filter A and the principal axis O*a*, for which the signal in the detector D has a maximum value.

To establish the polarization state of the emergent light from the anisotropic layer, a device schematically shown in Figure 1 is used. The light emitted by a monochromatic source S is converted into linearly polarized light by the polarizing filter P. By passing through the anisotropic layer, the light changes its polarization state, being established by the analyzer A, which is also a polarizing filter, together with the detector D. The converging lens CL1 and CL2 have the roles to transform the light beam emitted by the source S into a collimated one and to condensate the collimated light beam passing through the analyzer A onto the entrance slit of the detector, respectively.

The polarization state of light after AL depends on the phase difference Δ*ψ* introduced between the ordinary and extraordinary components of light (with their electric field intensity acting perpendicular and parallel to the optical axis, respectively):

(1) $$\Delta \psi =\frac{2\pi }{\lambda }\Delta nL.$$

In relation (1), *λ* is the wavelength of the monochromatic light, and *L* represents the thickness of the anisotropic layer crossed by light at normal incidence.

The linear polarized light crossing the anisotropic layer at normal incidence changes its polarization state as follows:

1. When Δ*ψ* = *mπ*, *m* = 0, 1, 2, …, the light remains linearly polarized, keeping its azimuth *α* (inclination of the electric field intensity relative to the optical axis of the anisotropic layer) if *m* is an even number or changing its azimuth to −*α* for an odd number *m*;
2. When $\Delta \psi =\left(2m+1\right)\frac{\pi }{2}$, *m* = 0, 1, 2, …, the light becomes elliptic polarized with its semiaxes parallel to the principal axes of the anisotropic layer (or circular polarized for azimuth angles being an odd number of *π*/4);
3. For any other cases, the light is elliptically polarized with its polarization ellipse rotated relative to the principal axes of the anisotropic layer. The rotation angle *θ* depends on the phase difference introduced by AL.

The changes in the polarization state of the light are used for estimating the value of the difference Δ*ψ* introduced by AL between the ordinary and the extraordinary components of light.

Two methods were used in the present research for estimating the linear birefringence of PET films: the ellipsometric method, allowing for establishing the linear birefringence value for monochromatic radiation, and the channeled spectrum method, estimating the linear birefringence and its dispersion for the entire visible range. The two methods use the P-AL-A system schematically represented in Figure 1.

In the case of the ellipsometric method, the P-AL-A system (see Figure 1) is illuminated by a monochromatic source in a collimating arrangement with a convergent lens (CL1 in Figure 1). A second convergent lens (CL2 in Figure 1) gives the image of the source S on the entrance slit of the detector D, in whose external electrical circuit the signal produced by the photoelectric effect is recorded.

**Figure 2:**
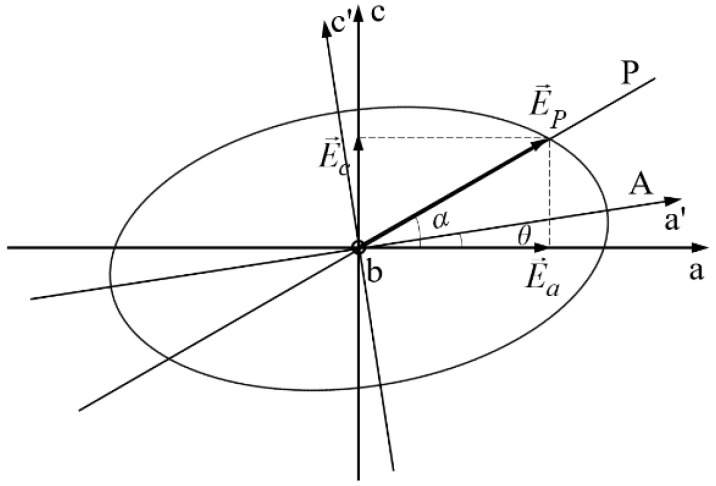
Relative positions of the transmission directions of the polarizing filters P and A in the principal plane O*ac* of the anisotropic layer.

If, at a complete rotation of the analyzer A around the propagation direction of the light, one obtains maxim and null values of the electric current in the detector, the radiation is linearly polarized, and the above condition (a) is accomplished. If two maxima and two minima different from zero are obtained for the same rotation of the analyzer A, the radiation is elliptically polarized, and the above condition (b) or (c) is fulfilled. When the transmission direction of P is parallel or perpendicular to the stretching direction of the polymer layer, the above condition (b) is accomplished. In this case, for azimuth angles with values quarters of *π*, one obtains circularly polarized radiation, and the detector D will indicate a constant value of the current in its external circuit when the analyzer A is rotated around the direction of the light propagation. If an azimuth angle *α* exists between the transmission direction of P and the principal axis of AL, the above condition (c) is fulfilled when the phase difference introduced by AL is *mπ* < Δ*ψ* < (2*m* + 1)π/2. In this case, one notes the angle *θ* between the transmission direction of the analyzer A and the principal axis of AL for which the current in the external circuit of the detector D is maximum. This case is described in Figure 2. Between the azimuth angle *α* and the angle *θ*, the next relation was established [39]:

(2) tg θ=tg αcos Δψ.

The phase difference between the ordinary and extraordinary components of light, introduced by passing through AL, can be determined with a precision limited by the periodicity of the trigonometric function cosine. For the case of the anisotropic layer with high thickness or high value of the birefringence, this imprecision can be eliminated by using two polymer foils with close values of thickness, *L*_1_ and *L*_2_, having their optical axes crossed (to minimize the phase difference at the first dial of the trigonometric circle), as it was demonstrated in [39].

The solutions of Equation (2) are

(3) $$\Delta \psi =\arccos\frac{\mathrm{tg}\theta }{\mathrm{tg}\alpha }+2k\pi ,\;k\in Z.$$

For the case 0 < Δ*ψ* < *π*/2, Equation (3) has a unique solution. To eliminate the imprecision of evaluation, two PET films cut from the same stretched foil and with close values of the thickness, *L*_1_ and *L*_2_, were used, having their stretching directions crossed. In this way, the ordinary ray emerging from the first film becomes an extraordinary ray in the second film, and the phase difference is diminished, according to

(4) $$\Delta \psi =\frac{2\pi }{\lambda }\Delta n\left(L_{1}-L_{2}\right).$$

The ellipsometric method [40] allows for the estimation of the linear birefringence for a monochromatic component from the visible spectrum range. To determine the linear birefringence and its dispersion for the whole visible spectrum range, the method of channeled spectrum is recommended. This method supposes the use of a white light source and a spectrophotometer as a detector. Two identical polarization filters having parallel transmission directions must be introduced in the compensation beam. In the measuring beam of the spectrophotometer, the P-AL-A system is introduced with crossed polarizing filters and the principal axis of the anisotropic layer at 45° relative to the transmission directions of P and A.

**Figure 3:**
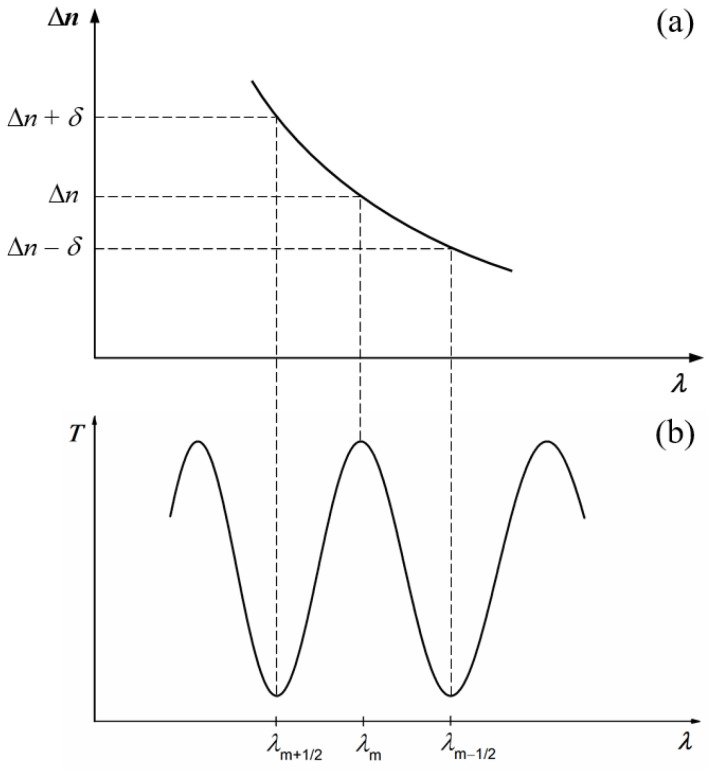
Variation in the linear birefringence with the wavelength (**a**), as well as two successive channels and the maximum between them in a channeled spectrum (**b**).

To establish the conditions for the channels’ appearance, let us consider the variation in the linear birefringence with the light wavelength (Figure 3a), as well as two successive channels, corresponding to the wavelengths *λ*_m+1/2_ and *λ*_m−1/2_, and the maximum between them, at *λ*_m_, in a channeled spectrum (Figure 3b).

According to Figure 3, the conditions of the appearance of two successive minima and the maximum between them can be written as follows:

(5) $$\{\begin{array}{l} \frac{2\pi }{\lambda_{m+1/2}}\left(\Delta n+\delta \right)L=\left(2m+1\right)\pi \\ \frac{2\pi }{\lambda_{m}}\Delta nL=2m\pi \\ \frac{2\pi }{\lambda_{m-1/2}}\left(\Delta n-\delta \right)L=\left(2m-1\right)\pi \end{array}.$$

The solutions of the equations system (5) for birefringence and its dispersion are

(6) $$\Delta n=\frac{1}{2L}\frac{\lambda_{m}\left(\lambda_{m-1/2}-\lambda_{m+1/2}\right)}{\lambda_{m+1/2}-2\lambda_{m}+\lambda_{m-1/2}},$$

(7) $$\delta =\frac{1}{2L}\frac{2\lambda_{m+1/2}\lambda_{m-1/2}-\lambda_{m}\left(\lambda_{m+1/2}+\lambda_{m-1/2}\right)}{\lambda_{m+1/2}-2\lambda_{m}+\lambda_{m-1/2}}.$$

The method of channeled spectra allows for the estimation of the birefringence and its dispersion for a large spectral range, by measuring only the wavelengths of the maxima and minima from the channeled spectrum and the thickness of the anisotropic layer.

The polarized ATR-FTIR measurements were performed using a Bruker Vertex 70 spectrometer (Bruker Optics GMBh, Ettlingen, Germany) by using a Golden Gate^TM^ ATR accessory, with a single bounce diamond crystal at the incidence angle of 45°. The wire grid polarizer was from PerkinElmer, Waltham, MA, USA. The polarized ATR spectra were achieved for a complete rotation cycle (0–360° polarization angles) and with steps of 15°. Each spectrum was an average of 128 scans at 2 cm^−1^ resolution, in the 4000–600 cm^−1^ wavelength range. Data analysis was performed using OPUS 6.5 software (Bruker Optiks GMbH, Ettlingen, Germany).

The polarized infrared spectra of elongated PET film were measured inside the polarization ellipse in two different experimental setups by placing the sample on the ATR crystal with the uniaxial stretching direction aligned parallel (machine direction) and perpendicular (transverse direction) to the IR beam direction, set at the *x* axis of laboratory coordinates. The transversal electric (TE) and transversal magnetic (TM) polarized waves have the electric field direction oriented perpendicularly and, respectively, parallel on the incidence plane of radiation. The dichroic measurements of stretched PET film used the TE wave, and the ATR absorbance ratios for the MD and TD configurations of a specific band were calculated. Depending on the spectral domain under analysis, the spectra were normalized to the skeletal ν(C=C) vibrations at 1503, 1410 cm^−1^ or to δ(CH)_o-o-p_ at 793 cm^−1^. The dichroic ratios of specific bands for initial PET have been calculated using the TE and TM waves for a fixed configuration of the sample. For the uniaxially stretched PET, the calculus of the dichroic ratio used the TE wave in the MD and TD configurations.

The dichroism spectrum was calculated as the difference between the 90° and 0° polarized spectrum, taking into consideration the difference in the relative intensity of the evanescent field for each polarization.

The structural factor spectrum *S*_0_ is a combination of parallel and perpendicular polarizations and represents the contribution from orientation changes only, and disregards the possible conformational changes produced by stretching [41,42]. The calculations use the formula $S_{0}=\left(S_{∥}+2S_{⊥}\right)/3$ [41,42].

## 3. Results and Discussion

The birefringence of stretched PET films was measured using the two methods described in the above section. The degree of stretching, *γ*, was determined by drawing a 1 cm segment on the unstretched film and measuring its length after stretching. The stretched PET film behaves as a uniaxial anisotropic layer, and its optical axis is located along the stretching direction.

### 3.1. Determination of the Stretched PET Films Birefringence by Ellipsometric Method

To measure the birefringence of the stretched PET films by the ellipsometric method, a device like the one described in Figure 1 was used. As a source, a sodium lamp was used, which provides yellow light at *λ* = 589.3 nm. The sample is illuminated by a collimated light beam with a diameter of 6 mm. The luminous flux is recorded using a Si-based photodetector with an amplifier, consisting of the spectral range of 390–1150 nm.

The light propagates perpendicular to the film’s surface, parallel with one principal axis of the anisotropic layer (direction Ob in Figure 2 and Figure 3). The azimuth angle *α* is fixed by rotating the polarizer P around the propagation direction. For a given value of *α*, the angle *θ* is measured by rotating the analyzer A around the same direction until the flux density (recorded by the detector D) reaches its maximum value. The rotation angles *α* and *θ* were measured with digital inclinometers fixed on the mounts of the polarizer P and analyzer A, respectively, with an accuracy of 0.01°.

Because of the rather high values of the PET film’s birefringence, two PET films cut from the same stretched foil, having close values of the thickness, and assembled with their optical axes crossed were used. In this case, the phase difference between the ordinary and extraordinary rays introduced by the anisotropic layer is reduced, it being proportional to the difference between the thickness values of the two PET films (see Equation (4)).

The results of the measurements, made for three values of the stretching degree, are given in Table 1, Table 2 and Table 3. The values given in these tables are the average values of ten measurements.

**Figure 4:**
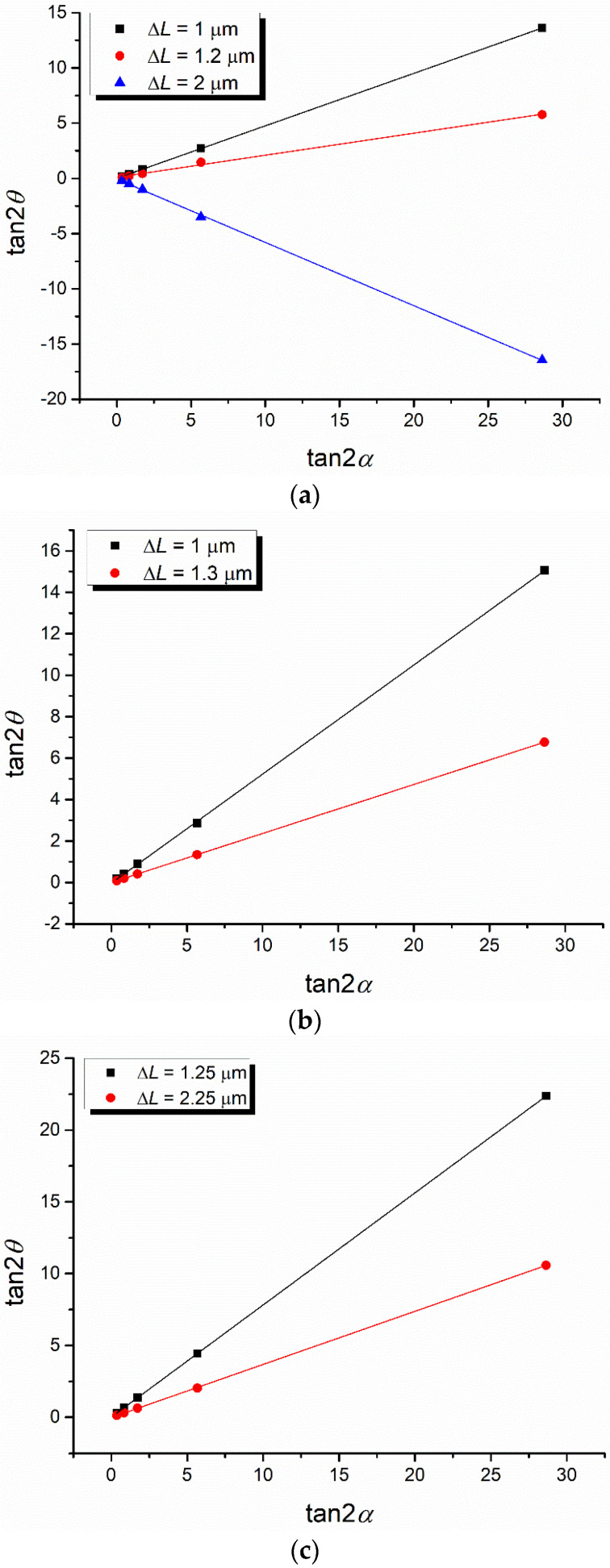
Variation in tan 2*θ* versus tan 2*α* for the PET films with the stretching degrees *γ* = 3.1 (**a**), *γ* = 2.4 (**b**), and *γ* = 1.66 (**c**).

According to Equation (2), cos Δ*ψ* is equal to the slope of the linear dependence between tan 2*θ* and tan 2*α*. Figure 4 shows this dependence for the data in Table 1, Table 2 and Table 3, respectively.

By linearly fitting the data in Figure 4, the phase difference Δ*ψ* between the ordinary and extraordinary rays can be obtained (according to Equations (2) and (3)), and then the value of the birefringence is determined using Equation (4). The obtained data are presented in Table 4.

As can be observed from Table 4, the birefringence is proportional to the stretching degree of the PET film.

### 3.2. Determination of the Stretched PET Films Birefringence by Channeled Spectra Method

For this method, a deuterium–tungsten halogen UV-Vis-NIR light source was used for the illumination of the PET samples, while the channeled spectra were recorded with an Ocean Optics QE65000 spectrometer (Ocean Insight, Orlando, FL, USA).

If the polarizing filters P and A are crossed and the principal axes of the PET film make an angle *π*/4 with the transmission directions of the polarizing filters, the transmission factor, defined as the ratio between the measured light flux and the light flux before the polymer film, is given by [40] as follows:

(8) $$T=\sin^{2}\frac{2\pi }{\lambda }\Delta nL.$$

**Figure 5:**
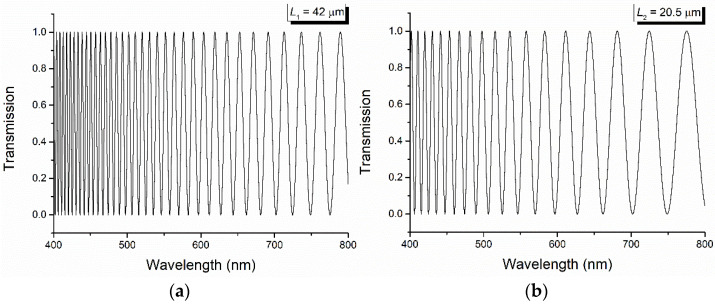
Channeled spectra recorded for two stretched PET films with stretched degree *γ* = 2.4 and the thickness *L*_1_ = 42 μm (**a**) and *L*_2_ = 20.5 μm (**b**).

**Figure 6:**
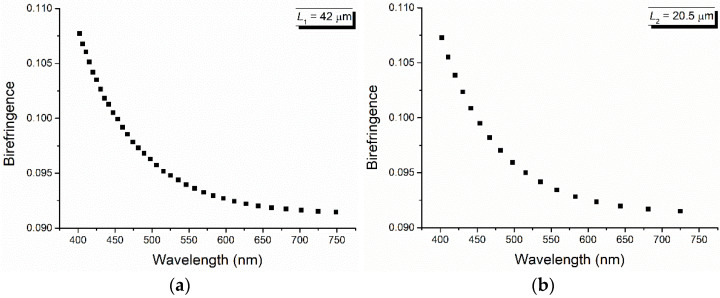
Birefringence calculated from the channeled spectra for two stretched PET films with stretched degree *γ* = 2.4 and the thickness *L*_1_ = 42 μm (**a**) and *L*_2_ = 20.5 μm (**b**).

**Figure 7:**
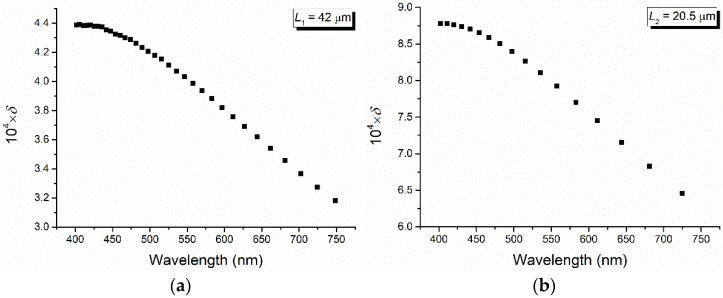
Dispersion of birefringence calculated from channeled spectra for two stretched PET films with stretched degree *γ* = 2.4 and the thickness *L*_1_ = 42 μm (**a**) and *L*_2_ = 20.5 μm (**b**).

The channeled spectra obtained for two stretched PET foils with *γ* = 2.4 and the thickness *L*_1_ = 42 μm and *L*_2_ = 20.5 μm, respectively, are shown in Figure 5. From these spectra, by reading the values of the wavelength corresponding to the maxima and minima and applying the relations (6) and (7) (similar results are also obtained by considering two maxima and the minimum between them), the values of the birefringence and its dispersion are obtained, as shown in Figure 6 and Figure 7.

From Figure 6, a decrease in the linear birefringence for the two stretched PET films (calculated by the channeled spectra method) with the increase in the wavelength is observed. Also, the dispersion of the PET birefringence decreases with the increase in the wavelength, and its results are shown in Figure 7. This late decrease is accentuated by high values of the wavelength, a region in which it becomes linear.

### 3.3. Infrared Spectral Analysis

Inherently, the uniaxial drawing of a PET film is accompanied by microstructural and conformational changes. The long macromolecular chains generally become oriented towards the drawing direction, but the specific orientation may differ between conformers. The monomeric unit of PET is characterized by several rotational isomers, each pair being specifically located in one chemical moiety and giving rise to specific infrared absorptions. The *trans* and *gauche* conformers of the ethylene unit are responsible for the ordered and disordered arrangement of PET chains, so the deformation vibrations of CH_2_ groups are usually employed to identify and quantify the crystalline and amorphous fractions of PET. The mutual arrangement of the ester carbonyl in either *cis* or *trans* position and two other *gauche* and *trans* isomers, which arise from a free rotation in the glycol O–CH_2_ bond, raise the number of possible conformations in the monomeric unit and complicate the infrared spectrum. The conventional ATR infrared spectroscopy catches the average population and orientation of these conformers, but a spectral differentiation of them could be made by means of polarized ATR-FTIR spectroscopy.

#### 3.3.1. General Remarks

**Figure 8:**
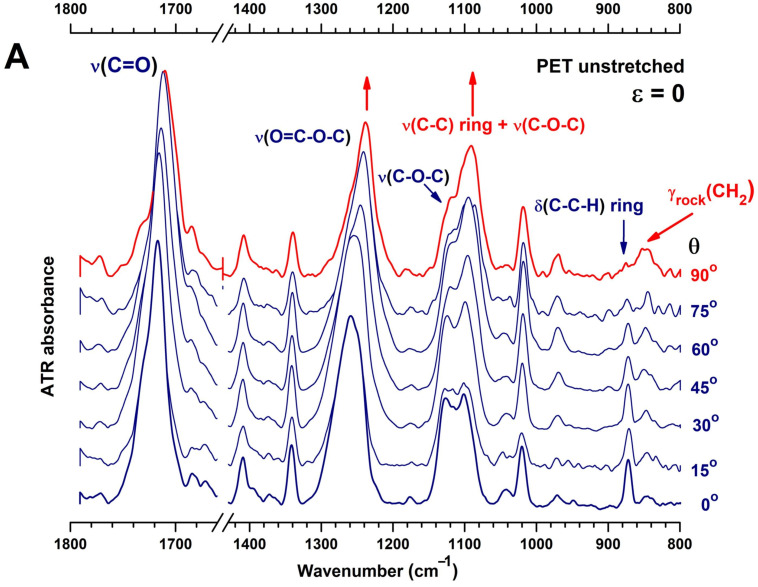

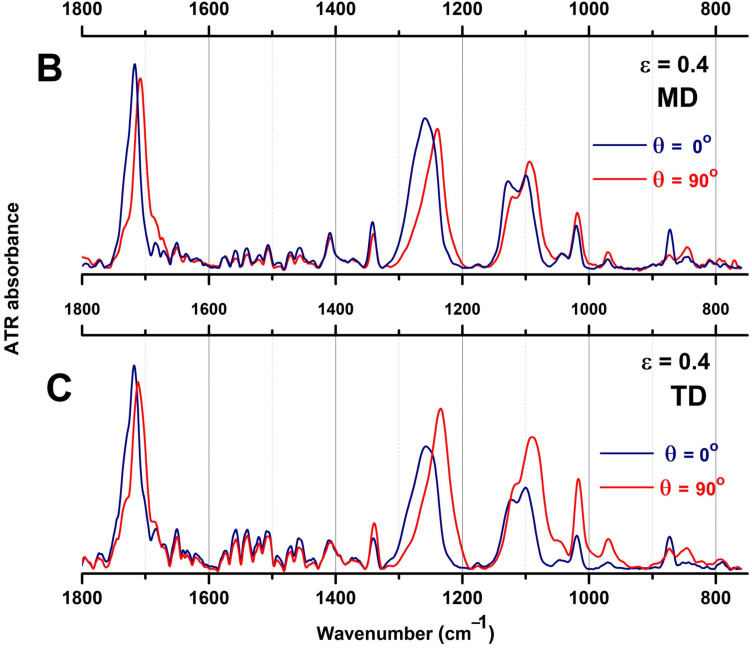
Polarized ATR-FTIR spectra in the fingerprint region of a commercial PET film, before and after stretching: (**A**) angle-resolved polarized ATR spectra of unstretched PET film for polarization angles between 0 and 90°; (**B**,**C**) parallel and perpendicularly ATR polarized spectra of the uniaxially stretched PET (*ε* = 0.4), recorded in the machine direction (MD) and transverse direction (TD), respectively. The arrows indicate ATR modes whose transition dipole moments are oriented parallel (red) and perpendicular (dark blue) to the ATR crystal and chain axis.

The polarized ATR-FTIR spectra of the initial PET shown in Figure 8A are selected from the spectra recorded by rotating the polarizer every 15° until a complete rotation is achieved. The strong ν(C=O), ν(O=C-O-C), [ν(C-C)_ring_ + ν(C-O-C)], δ(C-C-H)_ring_ and γ_rock_(CH_2_) vibrations observed around 1720, 1259, 1120, 872, and 845 cm^−1^ change their profile according to the polarization of light and show the native anisotropy of the unstretched PET film. The bands with perpendicular polarization (referenced to the ATR–sample interface) at 1717, 1685, 1341, 1258, 1126/1101, and 872 cm^−1^ seem to characterize a mixture of amorphous fraction and isolated *trans* sequences or possessing a low degree of order. The S_⊥_ spectrum resembles the TX spectrum obtained by Cole et al. by factor analysis [41]. The dominance of *gauche* conformers is indicated by the vibrations located at 1258 cm^−1^ (ester ν(O=C-O-C)), 1102 and 1042 cm^−1^ (glycol ν(C-O)) [42]. The *trans* conformers are indicated by the CH_2_ wagging vibration at 1341 cm^−1^. The in-plane δ(C-H)_ring_ peaking at 1020 cm^−1^ corresponds to a *trans* amorphous conformer [42]. The benzene δ(C-C-H)_ring_ mode at 872 cm^−1^ has the transition moment oriented perpendicular to the plane of the benzene ring, and this position belongs to the *trans* conformational isomer [43].

The spectrum recorded with parallel polarization contains the counterpart vibrations located at 1710, 1239, and 1091 cm^−1^, while the 872 cm^−1^ band (δ(C-C-H)_ring_) is greatly reduced and displays a very weak mode at 876 cm^−1^. This peak is very close to the 878 cm^−1^ position that Santoro et al. assigned to disordered regions [44]. Several more vibrations appear in *S*_II_: the “folding” band at 990 cm^−1^ that is associated with the crystalline lamellae [45], ν(O-CH_2_) at 969 cm^−1^, and γ_rock_(CH_2_) at 845 cm^−1^, the last two bands belonging to the *trans* configuration of ethylene glycol in ordered phase [41].

#### 3.3.2. Conformational Analysis

The in-depth analysis of the *S*_⊥_ and *S*_II_ spectra of the unstretched PET film allows us to differentiate several glycol and ester species. The ν(C=O) vibration is composed of the sub-bands at 1717 and 1685 cm^−1^ in the *S*_⊥_ spectrum and at 1735, 1710, and 1685 cm^−1^ in the *S*_II_ spectrum. The lower band at 1685 cm^−1^ corresponds to carbonyl groups in crystalline regions of “all-*trans*” structure [41,45,46,47], probably with a high density of packing and whose great redshift results from the strong intermolecular hydrogen bonds between the ordered chains. The lack of dichroism of the 1685 cm^−1^ band may arise from the uniform distribution of the crystal in the 3D space. The two maxima that selectively appear at 1717 cm^−1^ (*S*_⊥_) and at 1710 cm^−1^ (*S*_II_) seem to belong to two conformers of the terephthalate moiety, which interconvert via the rearrangement of the carbonyl group with respect to the phenyl ring. This mechanism has been used by Cole et al. to partially explain the crystallization process of amorphous PET [41]. Still, the position at 1710 cm^−1^ indicates a higher degree of packing of adjacent chains as compared to that at 1717 cm^−1^, which suggests that successive carbonyl groups framing the aromatic ring have a *trans* orientation and the whole fragment is in planar conformation [48]. The component at 1735 cm^−1^ is evident in both polarizations but is distinctive in parallel polarization as it is far away from the component at 1710 cm^−1^. As the carbonyls groups of PET are generally oriented perpendicularly to the backbone axis, the ester species at 1735 cm^−1^ seem to belong to chains having a random orientation in space, with a non-planar conformation of the terephthalate segment, so they belong to fully amorphous domains.

Similarly, the complex band centered on 1120 cm^−1^ displays different components as a function of light polarization, which suggests that each of them has a specific orientation to the sample–ATR crystal interface. Bahl et al. described this band as a complex combination of ether and aromatic ring modes, consisting of ν(C-O-C), δ(C-O-C), δ(C-C-O), and ν(C-C) vibrations [49]. Thus, in the *S*_II_ spectrum, the asymmetric band centered on 1096 cm^−1^ is mostly the ν(C-O-C) mode in a *gauche* configuration [46] and whose transition moment is oriented along the chain axis and along the interface. Its pair is observed in the *S*_⊥_ spectrum at 1102 cm^−1^, although Štokr et al. assigned this band to a δ(C-C-H)_ring_ mode [46]. The peak at 1126 cm^−1^ that appears only in the *S*_⊥_ spectrum combines mainly ν(C-C)_ring_ ring mode and ester ν((C=O)-O) mode, being characteristic of the ordered phase [45,46].

Application of uniaxial stretching stress of *ε* = 0.4 ($\varepsilon =\Delta l/l_{0}$) in the so-called machine direction (MD), slightly above the *T_g_* temperature of PET, resulted in absorbance variations that changed the shape of bands. The two sets of ATR-FTIR spectra were recorded in the MD and TD direction for a full rotation cycle of the polarizer. Figure 8B,C present the angle–resolved spectra for polarization angles between 0° and 90°. All spectra were normalized to the ring C=C stretching mode at 1410 cm^−1^. The first thing to notice is the reciprocal intensity variations in the ether modes centered on 1120 cm^−1^, broadening of the ν(C=O) vibration at 1717 cm^−1^, and decreasing of the chain folding band at 990 cm^−1^. The following spectral analysis of the stretched PET combines observations from the as-recorded spectra, dichroism spectra, and structural factor spectra.

#### 3.3.3. Dichroism and Structural Factor Spectra

**Figure 9:**
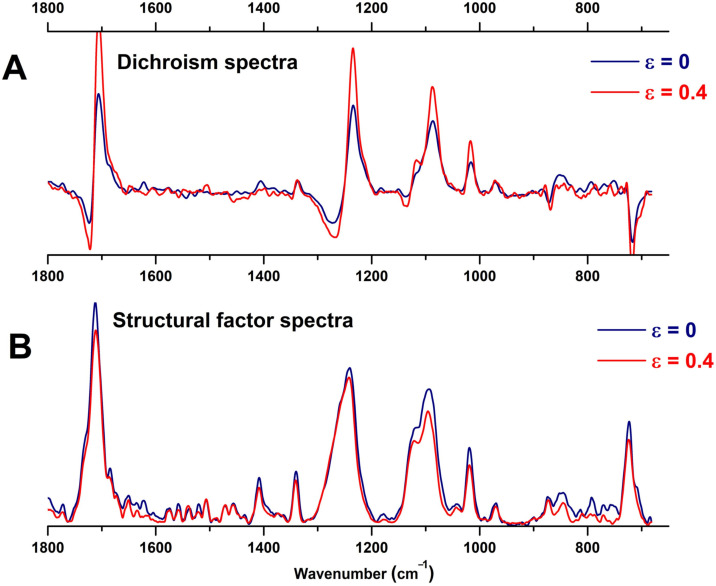

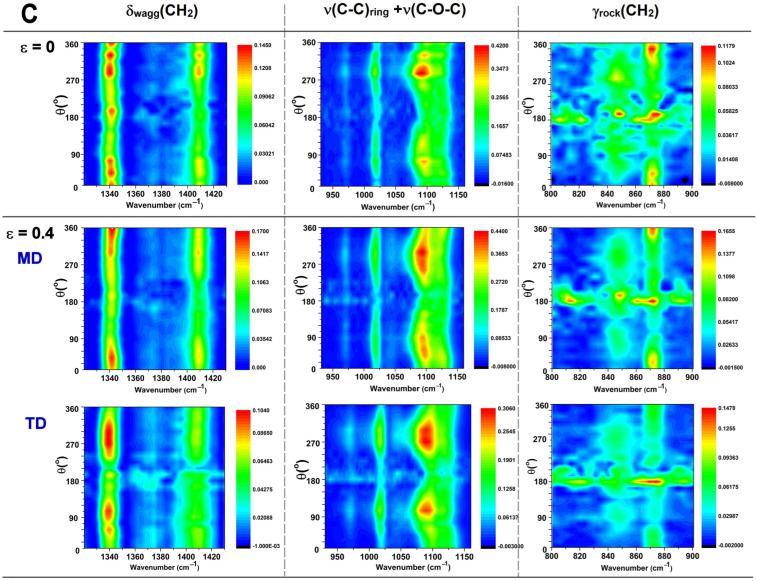
Advanced infrared spectral analysis of uniaxially stretched PET in comparison to native PET: (**A**) dichroism spectra (S_II_ − S_⊥_); (**B**) structural factor spectra; (**C**) polarized ATR spectra presented as color contour maps for the complete rotation of the polarizer, in three regions that correspond to vibrations of the ethylene glycol segment: ethylene wagging, ether stretching and ethylene rocking. The red arrows mark the vibrations with significant change after uniaxial elongation.

The dichroism spectra shown in Figure 9A highlight the peaks with evident dichroic character. The ester components at 1707 cm^−1^ (ν(C=O)) and 1234 cm^−1^ (ν(O=C-O-C), the glycol sub-bands at 1085 cm^−1^ (ν(C-O-C) and 969 cm^−1^ (ν(O-CH_2_)), and the benzene vibration at 1016 cm^−1^ (in-plane δ(C-H)_ring_) have the dipole moment oriented more or less close to the normal to the interface. The ester species that absorb at 1730 cm^−1^ and 1272 cm^−1^ have a perpendicular dichroism, with the transition moment oriented close to the ATR–film interface.

For the stretched PET, the enhanced dichroic character of the in-plane δ(C-H)_ring_ at 1018 cm^−1^ was attributed by Mecozzi and Nisini [50] and Santoro et al. [44] to a planarization of the benzene ring–ester frame. The ν(C=O) component, which in native PET appeared at 1707 cm^−1^, downshifted to 1706 cm^−1^ after stretching the film and has a narrow shape due to the same phenomenon. The glycol ν(O-CH_2_) band at 971 cm^−1^ has a new profile and covers two types of *trans* conformers: the amorphous *trans* at 975 cm^−1^ and crystalline *trans* around 969 cm^−1^ [44,50].

By using the *S*_⊥_ and *S*_II_ spectra recorded in MD mode, the “structural factor” spectrum $S_{0}=\left(S_{∥}+2S_{⊥}\right)/3$ was computed [41]. This approach eliminates the effects of the chain orientation and evidences the conformational changes or crystallization phenomena. The *S*_0_ spectrum of the stretched film resembles that of the initial film (Figure 9B). Large spectral changes are observed on the ester and glycol functionalities, and little changes in the wagging and rocking vibrations of ethylene groups, as the contour maps in Figure 9C show. Small increases in the γ_rock_(CH_2_) located now at 846 cm^−1^, which could be thought of as an increase in the fraction of isolated and quite ordered *trans* sequences [45,50], is in parallel with the enhancement of the ν(O-CH_2_) band at 970 cm^−1^ (redshifted from the initial 971 cm^−1^). Heuvel et al. showed that the 971 cm^−1^ band is redshifting under stress in PET yarns due to the uncoiling of disordered chains upon elongation and planarization in the ethylene glycol segment, followed by conformational *gauche*-*trans* change [50]. In our case, the chain elongation indeed produced the alignment of the glycol segment. But the *gauche-trans* isomerization took place to a small extent, and the fraction of converted *gauche* isomers is low.

#### 3.3.4. Dichroic Ratios and Molecular Orientation Function

The dichroism of an ATR absorption band is informative about the orientation of that specific vibration in reference to the macromolecular chain axis and to the ATR–film interface. The in-plane dichroic ratio *D* has been calculated as the ratio of normalized absorbances in the MD and TD modes by using the TE wave, that is $D=A_{∥}/A_{⊥}$. The reference peak was the aromatic ν(C=C) vibration at 1410 cm^−1^ for the 1500–1000 cm^−1^ domain and δ(CH)_o-o-p_ at 793 cm^−1^ for the 1000–750 cm^−1^ domain. The dichroic ratio *D* has been further used to determine Herman’s orientation function *f*, expressed in its simplified form as [42]:

(9) $$f=\frac{D-1}{D+2}\frac{D_{0}+2}{D_{0}-1},$$

where *D*_0_ is the dichroic ratio of a perfect oriented sample and has the expression

(10) $$D_{0}=2\cot^{2}\;\alpha .$$

The parameter *α* is the angle between the transition moment of a particular vibration mode and the macromolecular chain axis. The calculated values of *D* and *f* for the characteristic vibrations of PET film before and after stretching are listed in Table 5. For most of the PET vibrations, the *α* values are not known. However, Cunningham determined the tilt angle for the 872 cm^−1^ vibration of benzene as 85° [51], from which we determined the dichroic ratio as 0.28 in unstretched PET and 1.05 in oriented PET. The orientation function could be determined in our case only for the stretched PET (*f* = 0.02). In contrast with the literature [51,52], the values calculated by us for the dichroic ratios and for the orientation parameter are lower. The native anisotropy of the PET film, as it has been determined above in the general spectral analysis, may result from the manufacturing process of the roll.

The *trans* crystalline γ_rock_(CH_2_) at 848 cm^−1^ has the initial *D_u_* = 3.79, which decreased drastically after stretching to *D_s_* = 1.3. It appears that this band contains the absorption of a fraction of *trans* amorphous isomers that respond accordingly to the strain.

The dichroic ratio of the *gauche* γ_rock_(CH_2_) vibration at 896 cm^−1^ can estimate the orientational behavior of amorphous regions of PET upon uniaxially stretching. The *D_u_* and *D_s_* values are very close to each other, which is around 2, which suggests the *gauche* amorphous content of PET had almost zero orientation by stretching.

According to Spiby et al., the *α* value for the glycol ν(O-CH_2_) band at 975 cm^−1^ band is 34° [53], which leads to *D_u_* values of around 3.33 and 4.42, respectively, for the two species of *trans* isomers at 969 and 975 cm^−1^ (Table 5, Column 2). The *D_s_* values decrease after stretching the PET film, which is quite uncommon at low stress rates, and instead appeared at draw rates higher than 3.5 due to the development of a uniplanar structure [53].

Guèvremont et al. calculated *α* = 21.3° for δ(CH_2_)_wagg_ at 1340 cm^−1^ [52]. With this value, we obtained *D_u cryst_* = 0.85 and *D_u am_* = 0.98, which are much lower than those reported by Cole et al. [42] but are close to the values of Donelli et al. [54]. Uniaxial elongation of PET produced a small increase to ≈1.35 in stretched PET, and the increased dichroic ratios have been reported for both uniaxially and biaxially stretched PET [55]. The orientation factors *f_s_* in stretched PET are around 0.06 and 0.11, which are extremely close to the values calculated for the ν(O-CH_2_) vibrations at 969 and 975 cm^−1^, of 0.05 and 0.16, respectively.

#### 3.3.5. Microstructure of Stretched PET Revealed by Polarized ATR-FTIR Spectroscopy

**Figure 10:**
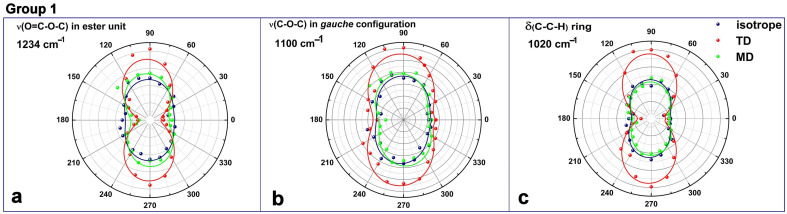
Polar representations of ATR-FTIR absorbance for the first group that defines the spectral changes (see text) as a function of polarization angle *θ*: (**a**) ester stretching at 1234 cm^−1^, (**b**) ether stretching at 1100 cm^−1^; (**c**) ring deformation at 1020 cm^−1^. The dots are the experimental data points that have been fitted with a cosine function. Experimental configurations: MD—green dots, TD—red dots, unstretched PET—dark blue dots.

To relate the orientation of the above sensitive vibrations to the polarization angle of the IR radiation, the infrared response is represented in anisotropy diagrams. The polar plot representations in Figure 10 give the angle-resolved normalized ATR absorbances of unstretched and stretched PET under two experimental configurations in reference to the unstretched, isotropic PET, measured every 15°. The experimental data points were fitted with a cosine function.

These anisotropy diagrams show that the ATR-FTIR spectral changes upon uniaxial stretching can be divided into three major groups:

- A change in intensity of the stretching vibrations of elliptically symmetric ester (1234 cm^−1^), ether (1100 cm^−1^), and ring modes (1020 and 872 cm^−1^);
- A change in symmetry for several deformation and stretching vibrations in CH_2_ groups (1341 cm^−1^), glycol (969/975 cm^−1^), and ester–glycol bridge (1120 cm^−1^);
- Intensity changes without symmetry change in the rocking CH_2_ vibrations at 845 and 896 cm^−1^, and in the carbonyl stretching at 1717/1710 cm^−1^.

The first group contains vibrations whose polar representations have a quadrantal elliptical anisotropy in the unstretched PET, according to Figure 10. The maximum IR intensity is towards 90° and 270°, showing the preferential alignment of the dipole moment parallel to the chain axis. In stretched PET, these vibrations show a conversion towards a 2-fold symmetry with a transition period of 180° and an increase in the magnitude of the components. Still, all these modes are characteristic of *gauche*/amorphous domains, and in addition, the ring modes are sensitive mainly to orientational effects. So, these variations do not indicate conformational changes but suggest that more chains became oriented to a certain degree.

**Figure 11:**
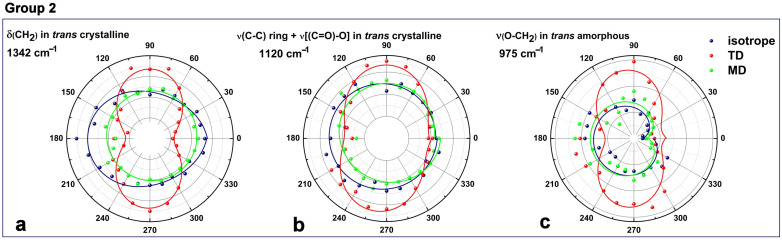
Polar representations of ATR-FTIR absorbance for the second group, which is characteristic mainly to *trans* sequences: (**a**,**b**) crystalline *trans*; (**c**) amorphous *trans*. The experimental and data analysis aspects are the same as those in Figure 10.

The second group, presented in Figure 11, consists mainly of δ_wagg_(CH_2_) at 1342 cm^−1^ (Figure 11a), ν(C-C)_ring_ + ν(C-O-C) at 1120 cm^−1^ (Figure 11b), and ν(O-CH_2_) at 969/975 cm^−1^ (Figure 11c); the initially non-symmetric (isotropic) distribution was changed to an elliptical/2-lobe symmetry. The symmetrical axis of the new structures after elongating the PET film is along the chain direction, an alignment that is best captured in the TD measurement mode. These vibrations mainly describe conformational changes in the ethylene–glycol segment and in isolated *trans* sequences that become ordered structures on a larger scale. Our findings share common points with observations recently evidenced via polarized Raman spectroscopy by González-Córdova et al. [56].

**Figure 12:**
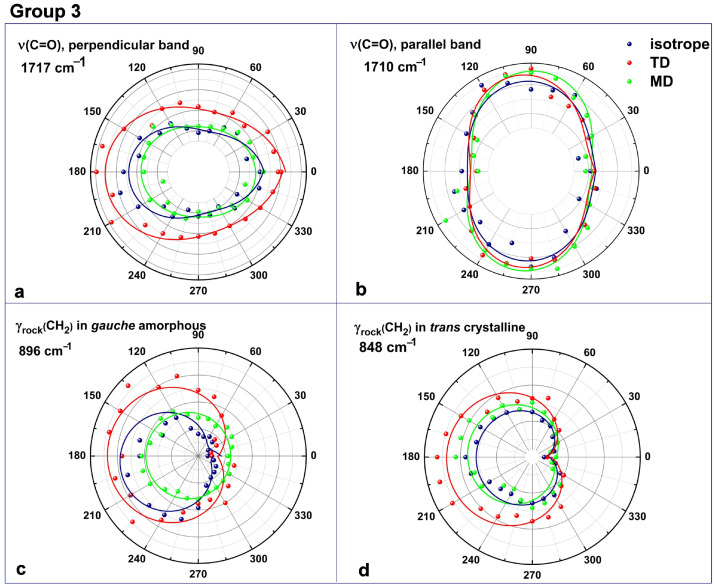
Polar representations of ATR-FTIR absorbance for the third group, whose spectral changes withdrawing are minimal: (**a**,**b**) the two components of the carbonyl stretching vibration; (**c**,**d**) methylene rocking vibrations in crystalline and amorphous domains, respectively. The experimental and data analysis aspects are the same as those in Figure 10.

The polar representation of the rocking CH_2_ vibrations in the third group is isotropic but non-centrosymmetric (Figure 12). The two carbonyl stretching vibrations at 1710 and 1717 cm^−1^ are oppositely oriented in the PET chain, and their initial symmetry is elliptical with a variation period of 180° (Figure 12a,b). As elongating the PET film did not lead to any change in the relative intensity of the 1710 cm^−1^ band, this fact confirms the above supposition about its origin in planar sequences from crystalline structures. The distribution of ν(C=O) at 1717 cm^−1^ shows that, despite the preferential orientation parallel to the chain axis, these carbonyls possess a high degree of freedom, and they belong to non-planar benzene–ester segments. By uniaxial stretching, these carbonyls become more oriented along the chain axis and are closer to the ATR crystal. Like the wagging motions, the rocking vibrations are neither parallel nor perpendicular polarized, as observed from the polarized ATR spectra. The ATR intensity of γ_rock_(CH_2_) mode (Figure 12c,d) uniformly maximizes with stretching but maintains the strange deviation as it was in native PET.

**Figure 13:**
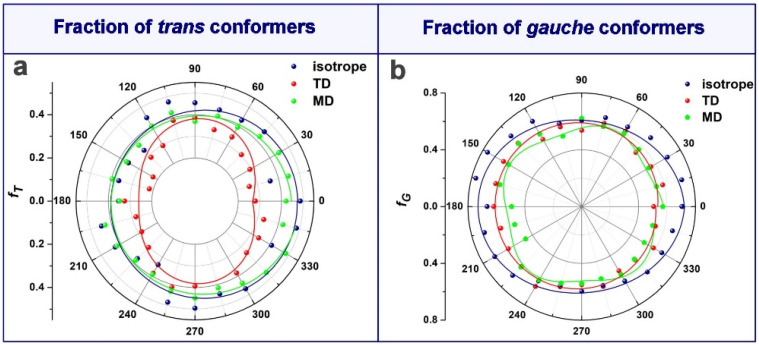
Anisotropy of the relative populations of *trans* (**a**) and *gauche* (**b**) conformers. The solid lines are fitted curves by a cosine function of the experimental data points.

Finally, the relative fraction of *trans* and *gauche* conformers, *f_T_* and *f_G_*, at the PET surface have been determined on the basis of their respective δ_wagg_(CH_2_) vibrations at 1341 and 1373 cm^−1^, respectively, according to the following relations [57]:

(11) $$f_{T}=\frac{OD_{1341}}{OD_{1341}+6.6OD_{1373}},$$

(12) $$f_{G}=\frac{6.6OD_{1373}}{OD_{1341}+6.6OD_{1373}}$$

where *OD* is the optical density of the respective ATR absorption band. The polar representations of *f_T_* and of *f_G_* in Figure 13 are a reflection of the whole analysis from above for the uniaxially stretched PET film. At the elongation rate of *ε* = 0.4 performed slightly above the glass transition, there is a small reduction in unordered *gauche* conformers, whose population generally did not respond to orientation. In parallel, the alignment of isolated *trans* sequences in the stretching direction occurred, and these newly ordered assemblies possess an elliptical symmetry.

## 4. Conclusions

Two methods were used to determine the linear birefringence of stretched PET films based on the change in the polarization state of linear polarized light crossing the anisotropic layer along one principal axis different from the stretching direction. The ellipsometric method allows the determination of the linear birefringence for monochromatic radiation, while the channeled spectrum method assures the estimation of the linear birefringence and also its dispersion on a large range of the visible spectrum for which the polarization filters can be used.

The linear birefringence of PET films with small values of the stretching degree (between 1.66 and 3.1), for which PET films can be considered as uniaxial materials, increases with the stretching degree and decreases with the increase in the wavelength of visible radiation.

The linear birefringence of PET films is a dispersive parameter whose dispersion also decreases with the increase in the wavelength of visible radiation.

The obtained values for the PET film’s birefringence and its dispersion are in good agreement with the values reported in the literature.

The FTIR studies of the microstructure of PET films and the configuration of some functional groups in PET samples emphasized the anisotropic properties of PET films by the dichroic ratio and ATR ratio for both stretched and unstretched PET samples.

## Figures and Tables

**Table 1:** Results of the ellipsometric measurements for PET films with *γ* = 3.1.

Δ*L* (μm)	*α* (°)	*θ* (°)	tan 2*α*	tan 2*θ*
1.0	10	4.80	0.36397	0.16914
	20	10.90	0.83910	0.39997
	30	20.00	1.73205	0.83910
	40	34.90	5.67128	2.71792
	44	42.90	28.63625	13.61741
1.2	10	2.62	0.36397	0.09171
	20	5.84	0.83910	0.20673
	30	11.52	1.73205	0.42530
	40	27.80	5.67128	1.46046
	44	40.09	28.63625	5.77736
2.0	10	84.50	0.36397	−0.19438
	20	76.91	0.83910	−0.49163
	30	67.30	1.73205	−1.01406
	40	52.98	5.67128	−3.49663
	44	46.74	28.63625	−16.44405

**Table 2:** Results of the ellipsometric measurements for PET films with *γ* = 2.4.

Δ*L* (μm)	*α* (°)	*θ* (°)	tan 2*α*	tan 2*θ*
1.0	10	5.20	0.36397	0.18353
	20	11.45	0.83910	0.42242
	30	21.10	1.73205	0.90674
	40	35.40	5.67128	2.87161
	44	43.10	28.63625	15.05572
1.3	10	2.55	0.36397	0.08925
	20	5.65	0.83910	0.19982
	30	11.20	1.73205	0.41217
	40	26.69	5.67128	1.34552
	44	40.80	28.63625	6.77199

**Table 3:** Results of the ellipsometric measurements for PET films with *γ* = 1.66.

Δ*L* (μm)	*α*(°)	*θ*(°)	tan 2*α*	tan 2*θ*
1.25	10	8.05	0.36397	0.28864
	20	16.80	0.83910	0.66440
	30	26.95	1.73205	1.37134
	40	38.65	5.67128	4.43735
	44	43.72	28.63625	22.36627
2.25	10	3.80	0.36397	0.13343
	20	8.51	0.83910	0.30611
	30	16.10	1.73205	0.62973
	40	31.90	5.67128	2.03227
	44	42.30	28.63625	10.57889

**Table 4:** Phase difference between the ordinary and extraordinary rays Δ*ψ* and the birefringence of PET films with different stretching degrees.

*γ*	Δ*L* (μm)	Δ*ψ* (°)	Δ*n*	$\bar{\Delta n}$
3.1	1.0	61.61786	0.10087	0.10342
	1.2	78.49929	0.10708	
	2.0	124.98626	0.10230	
2.4	1.0	58.23477	0.09533	0.09572
	1.3	76.32643	0.09611	
1.66	1.25	38.67712	0.05065	0.05017
	2.25	68.29857	0.04969	

**Table 5:** Peak positions, tilt angle *α*, dichroic ratios *D_u_*, *D_s_*, and Herman’s orientation functions *f_u_*, *f_s_*, for unstretched and stretched PET film (*ε* = 0.4).

Peak (cm^−1^)	*D_u_*	α (°)	*f_u_*	*D_s_*	*f_s_*	Conformation	Dichroism
845	3.79		--	1.28	--	γ_rock_(CH2), *trans* crystalline	π
872	0.28	85 ^1^	--	1.05	0.02	out-of-plane δ(C-C-H)_ring_ *gauche*	σ
898	2.33		--	2.16	--	*trans*	π
969	3.33	34 ^2^	0.47	1.14	0.05	ν(O-CH_2_), *trans*	π
975	4.42	34	0.58	0.61	0.16	ν(O-CH_2_), *trans* amorphous	π
1020	1.41	20 ^3^	0.14	1.13	0.05	in-plane δ(C-H)_ring_, *trans* amorphous	π
1100	1.27		-0.32	0.73	0.19	ν(C-O), *gauche*	π
1120	1.30			1.02	--	ν(C-C)_ring_ + ν(C-O-C), crystalline	π
1126	1.02		0.01	1.12	0.04	ν(C-C)_ring_ + ν(C-O-C), crystalline	π
1259	0.54	90 ^4^		1.07	0.02	ν(O=C-O-C)	σ
1234	1.64			1.41	--	ν(O=C-O-C)	π
1339	0.98	21.3 ^5^	−0.005	1.32	0.06	δ_wagg_(CH_2_), *trans* amorphous	π
1341	0.85	21.3 ^5^	−0.06	1.36	0.11	δ_wagg_(CH_2_), *trans* crystalline	π
1710	1.37			0.86		ν(C=O), crystalline	π
1717	0.63			0.87		ν(C=O), *trans* isolated	σ

^1^ Ref. [12], ^2^ Ref. [14], ^3^ Ref. [11], ^4^ perpendicular band, assumed value, ^5^ Ref. [13].

## Data Availability

The data presented in this study are available upon reasonable request from the corresponding author.
